# The high-affinity metal Transporters NRAMP1 and IRT1 Team up to Take up Iron under Sufficient Metal Provision

**DOI:** 10.1038/srep37222

**Published:** 2016-11-16

**Authors:** Loren Castaings, Antoine Caquot, Stéphanie Loubet, Catherine Curie

**Affiliations:** 1 Laboratoire de Biochimie et Physiologie Moléculaire des Plantes, Centre National de la Recherche Scientifique, Unité Mixte de Recherche 5004, Institut de Biologie Intégrative des Plantes, Montpellier, France

## Abstract

Iron (Fe) and manganese (Mn) are essential metals which, when scarce in the growth medium, are respectively taken up by the root high affinity transporters IRT1 and NRAMP1 in *Arabidopsis thaliana*. The molecular bases for low affinity transport however remained unknown. Since IRT1 and NRAMP1 have a broad range of substrates among metals, we tested the hypothesis that they might be functionally redundant by generating *nramp1 irt1* double mutants. These plants showed extreme Fe-deficiency symptoms despite optimal provision of the metal. Their phenotype, which includes low Fe and Mn contents and a defect of Fe entry into root cells as revealed by Fe staining, is rescued by high Fe supply. Using a promoter swap-based strategy, we showed that root endodermis retains the ability to carry out high affinity Fe transport and furthermore might be important to high-affinity Mn uptake. We concluded that NRAMP1 plays a pivotal role in Fe transport by cooperating with IRT1 to take up Fe in roots under replete conditions, thus providing the first evidence for a low affinity Fe uptake system in plants.

Iron (Fe) and manganese (Mn) are essential trace elements for living organisms. Because they can easily loose and gain electrons, these metals are energy-providing catalysers or cofactors for a range of enzymatic reactions. As such, they play important roles in oxidative stress responses, DNA synthesis or pathogen defence^1^. In plants especially, Fe and Mn are crucial for photosynthesis as core components of the photosynthetic machinery^2^,^3^. Like all transition metals however, Fe and Mn in excess become harmful because they catalyse the formation of reactive oxygen species through the Fenton reaction and the stimulation of the mitochondrial complex II, respectively^4^,^5^.

Plant Fe and Mn deficiencies occur in alkaline soils where most of the metals are present as insoluble complexes^6^,^7^,^8^. Fe deficiency is known to reduce chlorophyll synthesis and photosynthetic activity, leading to leaf interveinal chlorosis and decrease of biomass^9^,^10^. Mn deficiency symptoms, yet less documented, are also characterized by growth inhibition and leaf yellowing as well as tissue necrosis induced by the over-production of reactive oxygen species^3^.

On the contrary, Fe and Mn phytotoxicity can be reached in flooded areas and acidic soils where metals are readily accessible for plants^7^. Excess Fe causes growth arrest and necrotic spots on leaves known as bronzing symptom in rice^11^,^12^. Mn toxicity can also lead to chlorosis and necrotic leaf spots^13^. In higher plants, Fe and Mn are known to be antagonistic so that the excess of one of these metals systematically provokes a secondary deficiency of the other, thus reflecting the competition existing for their absorption^7^,^14^.

In non-grass plants, to which the model species *Arabidopsis thaliana* (Arabidopsis here after) belongs, Fe acquisition by Fe-limited roots relies on secretion of phenolic compounds by the root^15^,^16^,^17^,^18^, rhizosphere acidification by a membrane-bound proton ATPase that increases Fe solubility^19^, reduction of Fe (III) to Fe (II) at the root cell surface and subsequent Fe (II) import into the cell^20^,^21^. In Arabidopsis, this last step is mainly performed by the Fe deficiency-induced root plasma membrane transporter IRT1 (IRON-REGULATED TRANPORTER 1), which promotes high affinity Fe uptake under Fe-deficient conditions^22^,^23^. IRT1 has a broad range of substrates among divalent metals since it is able to complement yeast strains defective in Mn or zinc (Zn) uptake^24^ and since the *irt1-1* loss-of-function mutant fails to accumulate Mn, Zn and cobalt (Co) under Fe-deficient conditions^22^. The exact role of IRT1 in Mn, Zn and Co homeostasis has however not yet been investigated.

Recent studies with rice and Arabidopsis have shown the importance of metal transporters of the NRAMP (NATURAL RESISTANCE-ASSOCIATED MACROPHAGE PROTEIN) family in Mn uptake in plants^25^,^26^. NRAMP1 is the major high affinity Mn transporter in Arabidopsis as shown by the hypersensitivity of the *nramp1-1* mutant to Mn limitation and its inability to take up Mn in these conditions^25^. The expression of this plasma membrane transporter is upregulated in roots under Mn deficiency. Interestingly, *NRAMP1* is also induced upon Fe deficiency by FIT, the master regulator of the Fe deficiency response^27^,^28^ which suggests that it might play a role in Fe homeostasis as well. This hypothesis was further supported by NRAMP1 ability to restore *irt1-1* mutant Fe and Co uptakes, indicating that NRAMP1 too has a broad selectivity *in vivo*^25^.

Given the obvious yet poorly understood link existing between Fe and Mn uptakes, we sought to shed light on the respective contribution of IRT1 and NRAMP1 to Fe and Mn absorption by plant roots. A reverse genetics approach coupled with a promoter swap strategy allowed us to unravel the functional redundancy of NRAMP1 and IRT1 transporters for the uptake of these two metals under optimal growth conditions and revealed a possible root cell-specificity for Mn absorption.

## Results

### Knocking out both *IRT1* and *NRAMP1* has a dramatic impact on plant growth

**Figure 1:**
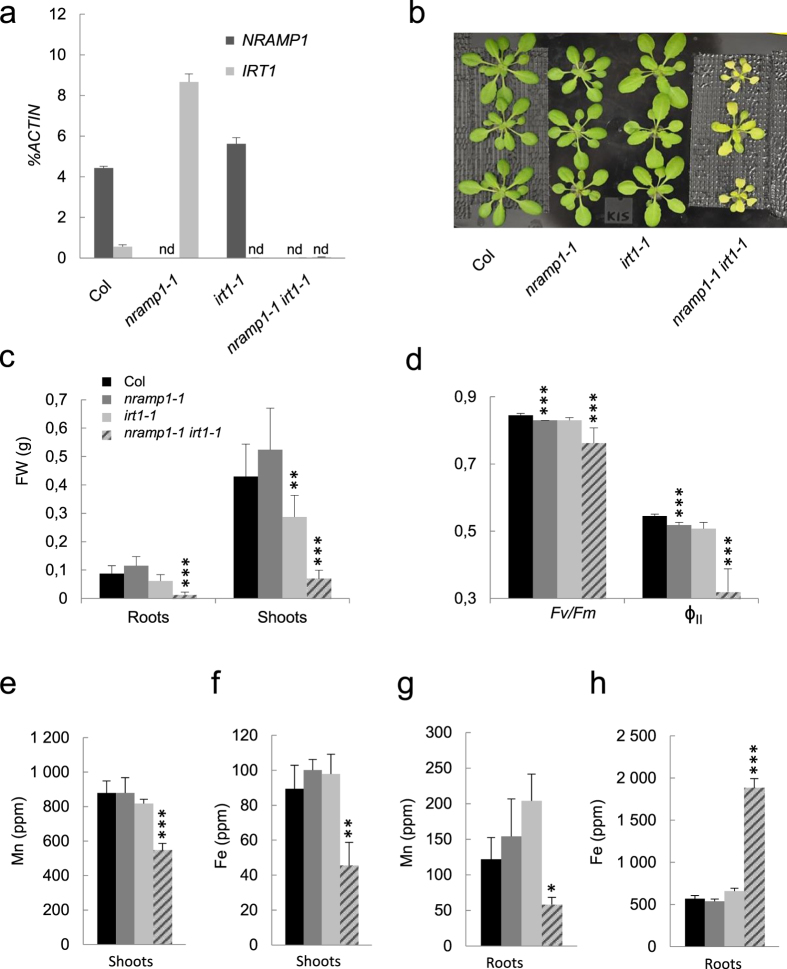
Phenotypic and elemental analyses of the *nramp1-1 irt1-1* double mutant. (**a**) *NRAMP1* and *IRT1* expression in *nramp1-1, irt1-1* and *nramp1-1 irt1-1* double mutant as compared to wild type Col-8 plants. RT-qPCR on RNA extracted from roots of 4 week-old plants grown in hydroponic cultures under standard (25 μM Fe, 20 μM Mn) conditions. Transcripts levels are expressed relative to those of the reference gene *ACTIN *± sd. (n = 3 technical replicates). nd, non-detectable. Two biological replicates of this experiment are shown in Supplementary Fig. S1. (**b**) 21 day-old plants grown as in (**a**). (**c**) Fresh biomass, (**d**) photosynthetic parameters (maximal quantum yield Fv/Fm and effective quantum yield ф_II_), (**e**) and (**f**) respectively shoot Mn and Fe contents, (**g**) and (**h**) respectively root Mn and Fe contents of 4 week-old plants grown as in (**a**). (**c** to **h**) share colour code legends. Mean ± sd. (n = 8 to 11 plants in (**c**); n = 4 to 5 plants in (**d**), n = 3 to 4 replicates of 8 to 10 plants each in (**e** to **h**)). Asterisks indicate values statistically different from those of Col plants (Student T-test, *P-value < 0.05, **P-value < 0.01, ***P-value < 0.001).

To assess a possible functional redundancy between IRT1 and NRAMP1 in Mn and/or Fe uptake, we first checked *IRT1* and *NRAMP1* expression in their respective mutants, namely *irt1-1* (Wassilewskija ecotype, Ws) and *nramp1-1* (Columbia ecotype, Col). Interestingly, *IRT1* was consistently overexpressed in *nramp1-1,* even under standard growth conditions (20 μM Mn, 25 μM Fe) (Fig. 1a and Supplementary Fig. S1). This result suggests that compensation mechanisms possibly hide Fe-related phenotypes in the *nramp1-1* mutant. In order to investigate this hypothesis, we crossed *irt1-1* and *nramp1-1* to generate a double *nramp1-1 irt1-1* mutant (see Methods). Among the F2 progeny, all homozygous plants for both *nramp1-1* and *irt1-1* mutations showed slow growth and a strong chlorosis (Supplementary Fig. S2). We therefore selected one of these knock out lines to further investigate the consequence of *NRAMP1* and *IRT1* simultaneous loss of function (Fig. 1a and Supplementary Fig. S1).

We opted for hydroponic cultures that enable control of the nutrients supply. When grown in that system under standard Mn and Fe supply (20 μM Mn and 25 μM Fe, see Methods), *nramp1-1* developed normally despite a small but significant reduction in the activity of the photosystem II (PSII) (Fig. 1b–d). In these conditions, *irt1-1* mutant only harbored a slight reduction in shoot biomass and accession-related morphological specificities such as bigger leaf blades and a wider expanded rosette similar to these of Ws ecotype (Fig. 1b,c and ref. 29). Our standard hydroponic medium provides therefore Mn and Fe in sufficient amounts for *nramp1-1* and *irt1-1* single mutants to grow nearly as well as wild type plants. In the same hydroponic conditions however, the double mutant developed similar growth defects than those previously observed in soil. Indeed, *nramp1-1 irt1-1* displayed a dramatically impaired growth of both shoots and roots (Fig. 1b,c) and a strong chlorosis associated with a poor photosynthesis efficiency as shown by the reduction of both the energy transfer within the PSII (*Fv/Fm*) and the light utilization (φ_II_) (Fig. 1b,d).

**Figure 2:**
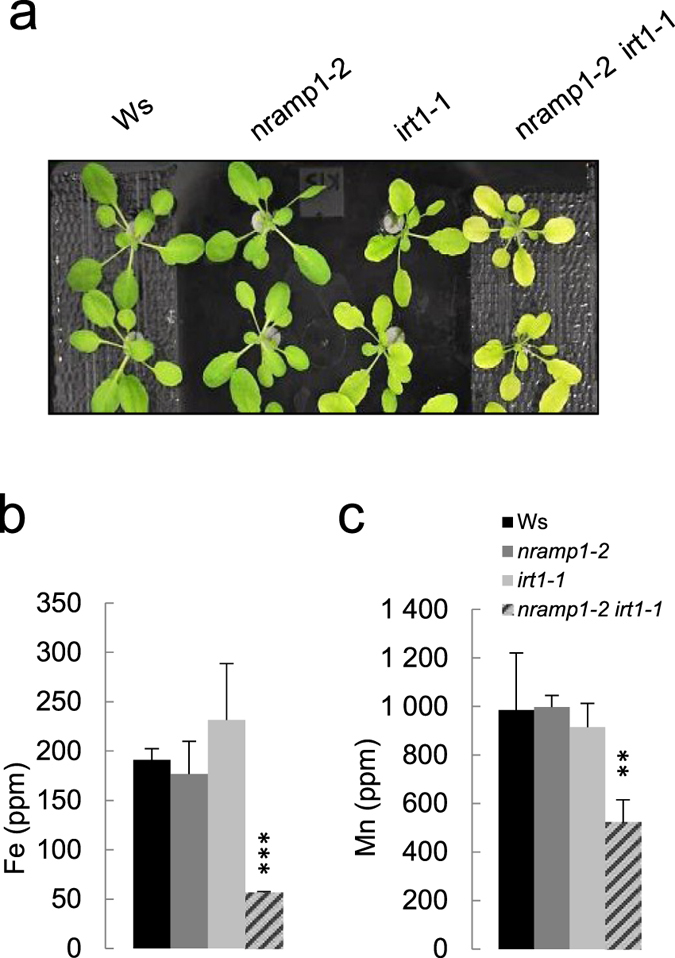
Phenotypic and elemental analyses of the *nramp1-2 irt1-1* double mutant. (**a**) 28 day-old plants grown in hydroponic medium containing 10 μM Fe and 25 μM Mn. (**b**) and (**c**) respectively Fe and Mn content in shoots of the plants shown in (**a**). Means ± sd. (n = 2 to 3 replicates of 3 plants each). Asterisks indicate values statistically different from those of Ws plants (Student T-test, *P-value < 0.1, **P-value < 0.05, ***P-value < 0.005).

To confirm this phenotype, we produced a second allele of *nramp1 irt1* double mutant using a new mutant allele of *NRAMP1* in the Ws ecotype pitched *nramp1-2* (Supplementary Fig. S3). The *nramp1-2* mutant, despite retaining 20% of the wild-type amount of *NRAMP1* transcripts, was hypersensitive to Mn deficiency, similar to the phenotype of *nramp1-1* (Supplementary Fig. S3). When grown hydroponically, *nramp1-2 irt1-1* plants harbored similar though milder phenotypes than those of *nramp1-1 irt1-1*. They were smaller and had paler leaves than *irt1-1* especially when challenged with limiting Fe supply (10 μM Fe, Fig. 2a, Supplementary Fig. S4). The difference in phenotype intensity between the two double mutant lines might come from the residual expression of *NRAMP1* from the *nramp1-2* allele. Also, different genetic backgrounds of the double mutants (Col/Ws for *nramp1-1 irt1-1* and Ws for *nramp1-2 irt1-1*) might be of influence on the phenotype severity. The latter explanation is however less likely since all the independent *nramp1-1 irt1-1* double mutant lines obtained from the initial cross harbored a strong phenotype despite their different Col/Ws allelic combinations (Supplementary Fig. S2).

Altogether these data suggest that NRAMP1 and IRT1 contribute to plant growth under optimal metal supply in a redundant manner.

### Fe and Mn levels are reduced in the two different *nramp1 irt1* double mutants

In order to further characterize the impact of inactivating both *IRT1* and *NRAMP1*, we measured metal content in *nramp1-1 irt1-1* double mutant plants and compared it to that of wild type plants and parental lines of the single mutants grown under standard metal supply (25 μM Fe, 20 μM Mn) (Figs 1e–h and 2b,c, and Supplementary Table S1). Among the tested metals (Zn, Cu, Mn and Fe), only Fe and Mn showed significant concentration changes in *nramp1-1 irt1-1* (Supplementary Table S1). Indeed, Fe and Mn contents in shoots were respectively 50% and 40% lower in the double mutant compared to the other genotypes (Fig. 1e,f). Similar results were also obtained in *nramp1-2 irt1-1* double mutant (Fig. 2b,c and Supplementary Fig. S4).

**Figure 3:**
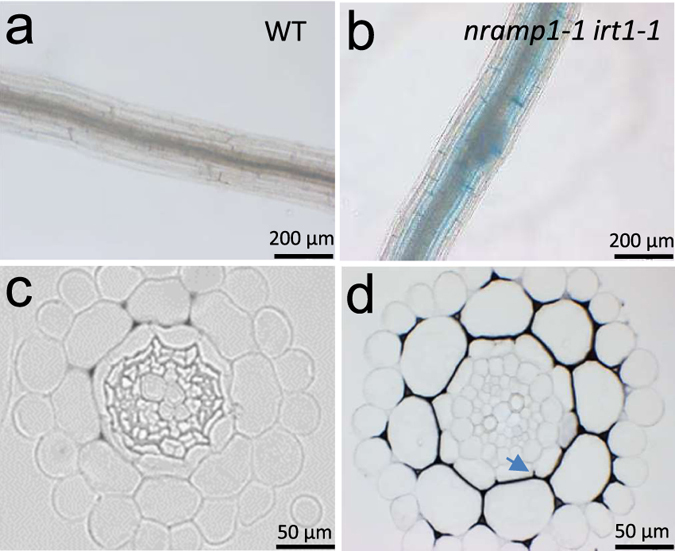
Fe accumulates in the apoplastic space of *nramp1-1 irt1-1* roots. (**a**) and (**b**) Perls staining of entire roots from 15 day-old wild type (WT) and *nramp1-1 irt1-1* seedlings grown *in vitro* in standard conditions. (**c**) and (**d**) Perls-DAB staining of thin root sections of WT and *nramp1-1 irt1-1* seedlings grown as in (**a**) A blue arrow in panel (**d**) highlights the interruption of the Perls-DAB labelling by the casparian strip.

A decrease in metal accumulation in shoots might be the consequence of impaired metal uptake by roots and/or poor metal translocation from roots to shoots. In order to identify which of these two mechanisms is defective in the double mutant, we measured metal content in roots. Mn concentration in *nramp1-1 irt1-1* roots was reduced twofold compared to wild type plants (Fig. 1g). The fact that Mn content was decreased in both shoot and root of *nramp1-1 irt1-1* plants strongly suggests that Mn uptake is impaired in the double mutant under optimal Mn concentration. Surprisingly, Fe was twice as much concentrated in the roots of *nramp1-1 irt1-1* compared to the other genotypes (Fig. 1h). This result was further confirmed by whole-mount roots stained with the Perls histochemical method that showed accumulation of Fe as a blue precipitate in the *nramp1-1 irt1-1* root (Fig. 3b). To refine the localization of such accumulated Fe in root tissues of the double mutant, wild type and *nramp1-1 irt1-1* root cross-sections were stained with the sensitive Perls-DAB method^30^. In wild type root sections, Fe deposits were only limited to the rim of a few cells of the cortex, the darker signal in the central cylinder corresponding to light refraction (Fig. 3c). In the root of *nramp1-1 irt1-1* however, Fe precipitates dramatically spread to the entire cortical cell layer (Fig. 3d). Furthermore, Fe accumulation was restricted to the apoplast surrounding these cells, a conclusion that is also supported by the observation that Perls-DAB labeling stops halfway through the endodermis where the casparian strip blocks apoplastic transport (Fig. 3d, arrow). This suggests that Fe cannot efficiently enter root cells owing to a defect of Fe uptake.

Taken together these data indicate that NRAMP1 and IRT1 act redundantly in the acquisition of Mn and Fe by the root, over a wide range of concentrations of these metals.

### Fe insufficiency is responsible for the poor growth of *nramp1 irt1* mutants

**Figure 4:**
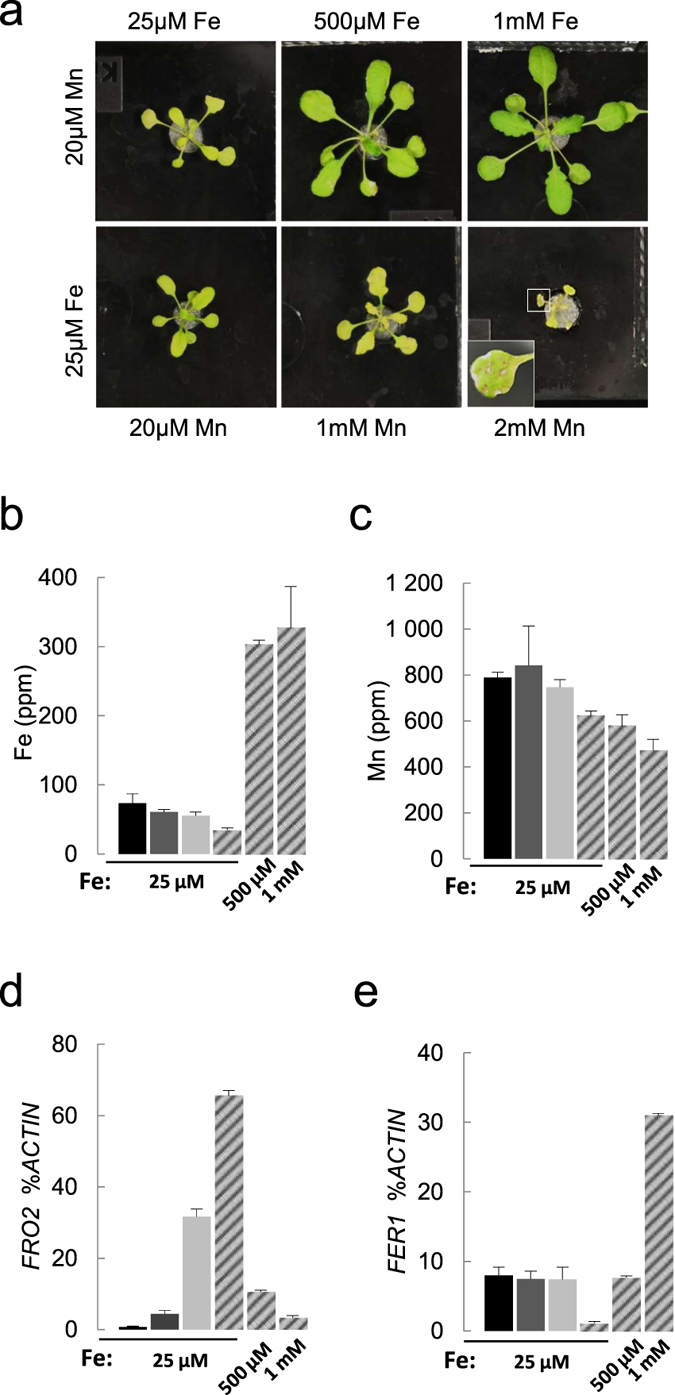
Fe, but not Mn, rescues the phenotype of *nramp1-1 irt1-1*. (**a**) Picture of 24 day-old *nramp1-1 irt1-1* mutants grown in hydroponic medium containing 20 μM Mn and a range of Fe supply (25 μM, 500 μM Fe, 1 mM Fe, top lane) or 25 μM Fe and a range of Mn supply (20 μM, 1 mM Mn, 2 mM Mn, bottom lane). The inset in the bottom right picture shows a close up of the leaf necrotic lesions. (**b**) and (**c**) respectively Fe and Mn concentration in shoots of plants grown as in (**a**). Wild type plants (black), *nramp1-1* (dark grey), *irt1-1* (light grey) and *nramp1-1 irt1-1* mutants (stripes). Means ± sd. (n = 3 replicates of 8 to 12 plants each). (**d**) and (**e**) Respectively *FRO2* and *FER1* expression in roots of the plants shown in (**b**) and (**c**). Transcripts levels were measured by qRT-PCR relative to those of the reference gene *ACTIN* ± s.d. (n = 3 technical replicates).

We then wanted to know which of the Fe and/or Mn deficiency observed in the *nramp1 irt1* double mutant is the most limiting factor for its growth and development. We therefore tested whether growth of the *nramp1-1 irt1-1* double mutant could be restored by increasing Fe or Mn supply. Interestingly, only Fe supplementation significantly improved growth (Fig. 4a). Under high Fe supply (500 μM or 1 mM), growth of *nramp1-1 irt1-1* was completely rescued and leaf chlorosis fully disappeared (Fig. 4a). Such a situation was not observed when Mn concentration was increased to 1 mM or 2 mM under constant Fe supply (25 μM). Instead, *nramp1-1 irt1-1* developed Mn excess symptoms including growth defect, leaf yellowing and necrotic spots (Fig. 4a).

Under high Fe supply conditions that revert *nramp1-1 irt1-1* phenotype, Fe content in leaves of the double mutant increased dramatically to a level exceeding more than threefold that of wild type plants grown in standard conditions, while Mn content slightly decreased (Fig. 4b,c). This result further strengthens the conclusion that despite being reduced in the double mutant, Mn concentration does not limit its growth. We concluded that Fe deficiency alone is responsible for *nramp1-1 irt1-1* poor growth under normal metal supply.

The Fe status of the double mutant under standard Fe supply was evaluated at the molecular level by monitoring root expression of the two iron homeostasis genes *FRO2* and *FER1* that respond to Fe availability in an opposite manner (Fig. 4d,e). When Fe is scarce, *FER1* expression decreases in order to free the pool of immobilized Fe^31^. *FRO2* is a direct marker of Fe deficiency in Arabidopsis and its expression goes up to increase the ferric reductase activity at the root surface and maximize Fe absorption^19^. Under standard Fe supply (25 μM), *FRO2* was not significantly deregulated in *nramp1-1* but was overexpressed in *irt1-1,* consistent with the *irt1-1* mutant being slightly affected in our standard hydroponic conditions (Figs 1c and 4d). In the *nramp1-1 irt1-1* double mutant, *FRO2* expression was further deregulated, showing a twofold higher expression compared to the *irt1-1* single mutant, thus confirming that the double mutant experiences a stronger Fe starvation (Fig. 4d). An over-induction of *FRO2* was also observed in the *nramp1-2 irt1-1* mutant, carrying a *NRAMP1* knock-down allele, when grown under limiting Fe supply, especially at 10 μM Fe where Fe content is the most reduced in the double mutant (Supplementary Fig. S4). As expected, supplementation of the double mutant with high Fe repressed *FRO2* expression. Interestingly however, whereas *FRO2* expression in wild type plants is minimal at 25 μM Fe supply, as much as 1 mM Fe are required to shut *FRO2* down in the double mutant (Fig. 4d). Consistently, we found the opposite situation with *FER1. FER1* mRNA levels were extremely low in *nramp1-1 irt1-1* grown in standard 25 μM Fe conditions compared to all the other genotypes, thus supporting our previous finding that Fe does not adequately penetrate root cells in these plants. In the double mutant, reversion of *FER1* expression to a level equivalent to wild type plants grown in standard medium required up to 500 μM Fe supplementation, which again points to a defect of Fe entry into root cells (Fig. 4e). Taken together our results show that poor growth and chlorosis symptoms observed in *nramp1 irt1* double mutants under Fe and Mn replete conditions are caused by extreme Fe deficiency due to impaired Fe acquisition by root cells.

### Assessment of *NRAMP1* and *IRT1* specificity of transport in various root territories by promoter-swapping

**Figure 5:**
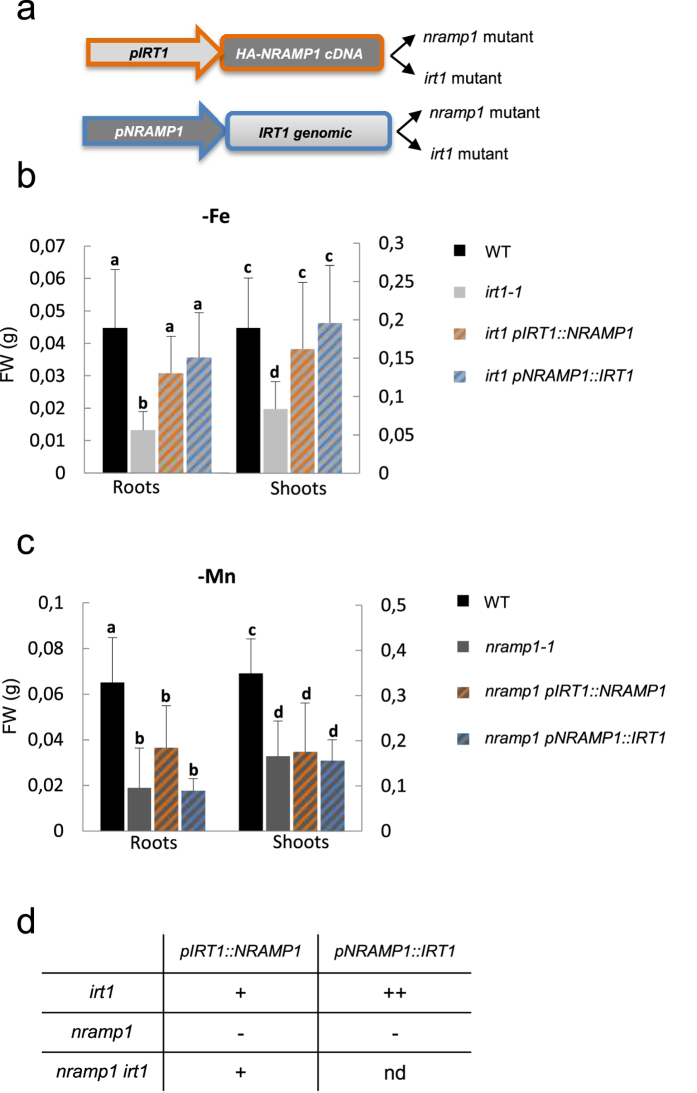
Complementation of *nramp1-1* and *irt1-1* mutants by promoter swap constructs. (**a**) Promoter swap design. (**b**) Complementation of *irt1-1* hypersensitivity to iron deficiency (-Fe) by promoter swap constructs was assessed by measuring the fresh weight (FW) of roots and shoots of 3 week-old plants grown 2 weeks in standard hydroponic conditions and one more week with no added iron. (**c**) Complementation of *nramp1-1* hypersensitivity to manganese deficiency (-Mn) by promoter swap constructs was assessed by fresh weight (FW) measurement of roots and shoots of 3 week-old plants grown in hydroponic conditions with no added manganese. In (**b**) and (**c**) Mean ± sd (n = 6 to 12 plants). Values that are not statistically different are marked with the same letter (ANOVA, α risk 5%). (**d**) Summary of the complementation results from (**b**), (**c**) and Supplementary Fig. S6. (++) optimal complementation, (+) good complementation, (−) absence of complementation, (nd) not determined.

qPCR data and promoter-GUS studies have established that *IRT1* and *NRAMP1* have expression territories that are both overlapping and distinct^22^,^25^. *IRT1* is mainly expressed in root epidermis and cortex of the absorption zone and in root hairs while *NRAMP1* expression is located more internally in cortex and endodermis from the root tip up to the mature zone as well as in the root meristematic zone^22^,^25^. *IRT1* and *NRAMP1* are also expressed at very low levels in shoots where they conserve a slight induction by Fe and Mn deficiency respectively^22^,^25^ (Supplementary Fig. S5). In order to test the importance of these expression patterns for the physiological function of NRAMP1 and IRT1, we carried out a promoter swap strategy. To that aim, *pIRT1::NRAMP1* and *pNRAMP1::IRT1* constructs were generated and expressed into both *nramp1* and *irt1* mutants (Fig. 5a, see Methods).

To examine the possibility that the expression territory of *NRAMP1* has the potential to carry out optimal iron acquisition in Fe-deficient roots, we tested whether *IRT1* expressed under the control of the *NRAMP1* promoter could rescue *irt1* hyper-sensitivity to Fe deficiency. When the *irt1 pNRAMP1::IRT1* transgenic line was grown hydroponically under Fe deficiency, growth of the *irt1* mutant was essentially rescued (Fig. 5b and Supplementary Fig. S6). Thus, despite *NRAMP1* promoter activity being very weak compared to *IRT1* in Fe deficient conditions (Supplementary Fig. S7), it is able to drive sufficient expression of *IRT1* in the adequate location to ensure high affinity Fe uptake by the root. As previously reported^25^, the *irt1* phenotype is also rescued by *pIRT1::NRAMP1* (Fig. 5b), indicating that NRAMP1 is able to transport Fe when strongly expressed in root epidermis and cortex under the control of the *IRT1* promoter. This result was further supported by the observation that *pIRT1::NRAMP1* is also able to rescue the Fe-deficiency phenotype of *nramp1-1 irt1-1* (Supplementary Fig. S6). Since the plant can be efficiently fed when Fe uptake occurs exclusively in the territories of expression of *NRAMP1* and since NRAMP1 can functionally replace IRT1, we concluded that NRAMP1 has the capacity to contribute to Fe acquisition by the root.

Next we asked whether endodermal expression of NRAMP1 is important for its high-affinity Mn transport activity. To that aim, we tested whether *NRAMP1* expressed under the control of the *IRT1* promoter, which is only active in root outer cell layers excluding endodermis, could rescue *nramp1-1.* Interestingly, we found that *pIRT1::NRAMP1* is unable to restore the tolerance of *nramp1-1* to Mn-deficiency (Fig. 5c). Because the functionality of this construct was validated by its ability to successfully complement *irt1-1* (Fig. 5b), we concluded that endodermis is essential for high-affinity Mn acquisition by the root. Since root tip also shows differential *IRT1* and *NRAMP1* expression, it is also possible that root tip plays an important role in Mn uptake under Mn starvation conditions.

At last we evaluated whether IRT1 has the potential to carry out high affinity Mn uptake. To that aim we tested whether the expression of *IRT1* under the control of the *NRAMP1* promoter was able to complement *nramp1-1* hypersensitivity to Mn deficiency. We found that *pNRAMP1::IRT1* failed to rescue growth of *nramp1-1* under Mn deficiency (Fig. 5c and Supplementary Fig. S6). Since this construct is functional as shown by its ability to efficiently rescue the *irt1* phenotype (Fig. 5b), and since *IRT1* transcripts level in this line is high (Supplementary Fig. S7b), we concluded that IRT1 is unable to efficiently take up Mn in the high affinity range. We cannot however exclude the possibility that IRT1 protein is down-regulated at the post-transcriptional level when ectopically expressed in the root.

## Discussion

In an effort to assess the respective contributions of IRT1 and NRAMP1 to Fe and Mn acquisition by the plant, we generated *irt1 nramp1* double knockout lines that display unexpectedly strong Fe-starvation symptoms even in the absence of Fe limitation (Fig. 1). These mutants harbor dramatically low Fe content in the leaves. Moreover, *FRO2* upregulation and *FER1* downregulation in the root reveal that root cells of the double mutants are also Fe depleted. This phenotype is consistent with the double mutants being unable to properly intake Fe from the medium (Figs 1 and 2 and Supplementary Table S1), which also fits with the strong deposition of Fe in the apoplastic space of the outer layers of the root (Fig. 4). We therefore concluded that NRAMP1 and IRT1 play a redundant function in the conditions of sufficient Fe concentration in Arabidopsis.

The function of NRAMP1 in Fe acquisition was previously deduced from its FIT-dependent regulation and from its ability to partially rescue both the yeast Fe uptake mutant *fet3∆ fet4∆* and the Arabidopsis *irt1-1* mutant^25^,^27^,^28^,^32^. However, given that *nramp1* loss of function mutants fail to display detectable iron homeostasis defects, it was still unclear if and how the NRAMP1 transporter could contribute to Fe entry into the plant. Here we demonstrate that NRAMP1 is part of the low affinity Fe transport system in Arabidopsis. Fe and/or Mn deficiency could be the cause of the severe phenotype of the *nramp1-1 irt1-1* double mutant given that IRT1 and NRAMP1 are able to transport both metals^22^,^25^. However, the observation that only high Fe-, but not high Mn-, supply can rescue the double mutant indicated that Fe deficiency alone is responsible for the poor growth observed (Fig. 4). It therefore confirmed that in the absence of IRT1, the activity of NRAMP1 is an absolute requirement, at least in the “replete range” of Fe concentrations.

IRT1 has been well established as the major high affinity Fe transporter at the root surface in Arabidopsis^22^,^33^. Remarkably, our work reveals the yet undescribed activity of this transporter in the low affinity range. The chlorotic phenotype of the *nramp1-1 irt1-1* double mutant under Fe-replete conditions (25 μM Fe, Fig. 1b) strongly resembles that of the *irt1-1* single mutant grown under Fe-limited conditions^22^,^33^ and fully disappears when the plants are provided with very high Fe supply (500 μM to 1 mM Fe, Fig. 4a). These data and our previous work^22^ demonstrate therefore that IRT1 transports Fe within a wider range of concentrations than previously proposed, and enables us to conclude that in addition to being the major contributor of Fe uptake under Fe limiting condition, IRT1 also shares Fe transport activity with NRAMP1 when Fe availability comes close to Fe replete conditions.

Finally, the finding that as high as 1 mM Fe successfully rescues *nramp1-1 irt1-1* chlorosis and growth defect suggests the existence of an alternative transport pathway to IRT1 and NRAMP1 operating in the very low affinity range. Whether this very low affinity system is an active transporter or involves simple diffusion remains an open question.

This study also highlights interesting facts about root Mn acquisition. We found the Mn content of the *nramp1 irt1* double mutants to be markedly reduced under non-limiting Mn supply compared to each single mutant (Figs 1e,g and 2b, Supplementary Table S1, Supplemental Fig. S4). Aside from Fe, IRT1 can take up several other metals including Mn but the physiological relevance of this broad range specificity has remained unclear and challenged by the fact that only Fe supplementation can rescue the *irt1* mutant^22^. From the dramatic defect of Mn content of the *nramp1 irt1* double mutant, it is now clear that both IRT1 and NRAMP1 contribute to root low affinity Mn uptake. A second conclusion is that NRAMP1 has a wide affinity range for Mn, from limiting to optimal concentrations, a feature that it shares with rice OsNRAMP5 whose corresponding loss-of-function mutant cannot take up Mn regardless of its availability^26^. Unlike Fe however, high Mn supply cannot rescue *nramp1-1 irt1-1* growth defects (Fig. 4) suggesting that the double mutant does not suffer from Mn deficiency. This assumption is strengthened by our finding that *nramp1-1 irt1-1* plants were fully rescued by high Fe supply even though this treatment induced a stronger reduction in leaf Mn content (Fig. 4). This suggests that such low Mn concentrations likely remain above the minimal amount required to meet plant needs^1^.

This study points to an important function of the root endodermis in high affinity Mn uptake with the observation that NRAMP1, which is strongly expressed in endodermis^25^, is unable to rescue the *nramp1* mutant when only expressed in the epidermis and cortex under the control of the *IRT1* promoter (Fig. 5). Also in relation with its expression in inner cell layers of the root, an open question remained whether besides its role in metal acquisition, NRAMP1 could also act in root-to-shoot translocation of these metals. We found that Fe is retained in the root of the *nramp1-1 irt1-1* mutant but rather than a defect of translocation, root Fe content was mostly constituted by a large pool of apoplastic Fe surrounding cortical cells, pointing to an inhibition of Fe entry into the root rather than impaired translocation to the shoot. Shoot-to-root Mn ratio is conserved in *nramp1-1 irt1-1*, an additional indication that Mn translocation is not perturbed in the mutant.

Finally, this work raises the question of the interaction between Fe and Mn metabolisms in plants. As a general mechanism, Fe-deficient plants take up large amounts of metals including Mn. Both metals seem to take routes involving common transporters and as such, they compete with each other for entering into the plant as well as probably when crossing intracellular membranes. Whether this competition is biologically relevant is not understood. The *nramp1 irt1* mutants are depleted in both Fe and Mn. Although we assign its macroscopic phenotype mainly to the lack of Fe, a possibility exists that the strength of its phenotype is caused by the double deficiency that this plant experiences. Gene expression profiling of Arabidopsis plants experiencing double deficiency as well as of the *nramp1 irt1* mutant might reveal the existence of metabolic pathways relying on the combined presence of Fe and Mn and provide clues on the molecular determinants of a low affinity Fe transport system.

**Figure 6:**
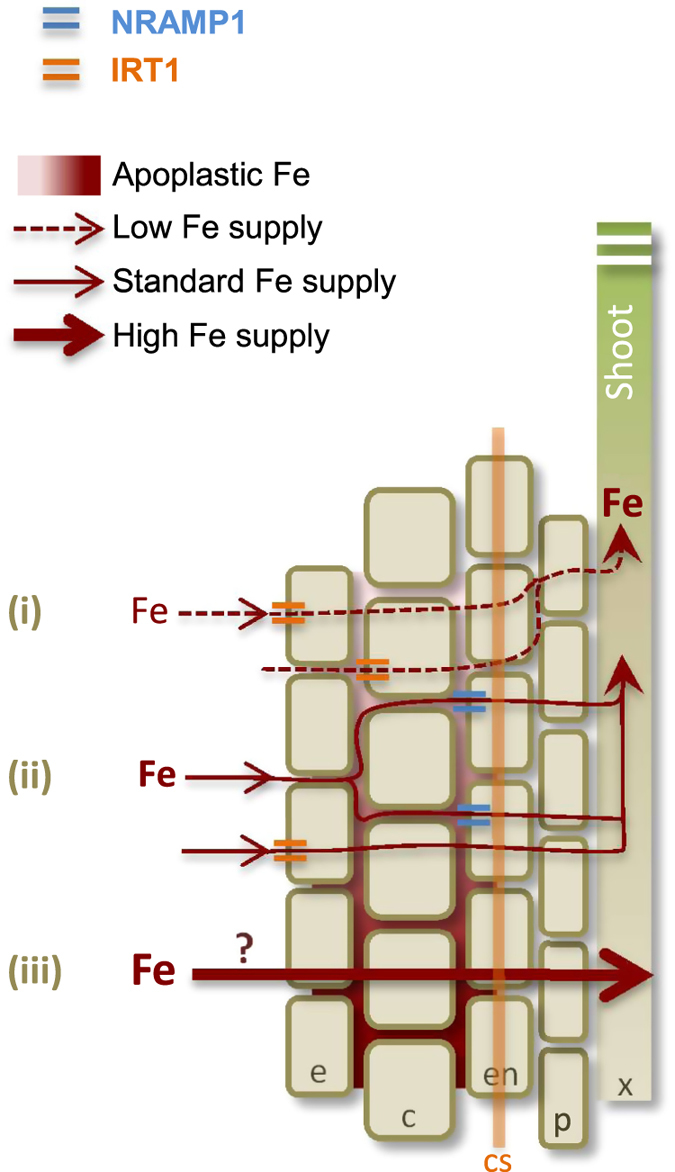
Hypothetical model of Fe acquisition systems in Arabidopsis root. (**i**) Upon Fe deprivation (<1 μM, dashed arrow), the IRT1 transporter mediates high affinity Fe uptake into epidermis and cortex. Loss of function of IRT1 provokes Fe deficiency symptoms but only in response to Fe limitation. (**ii**) Under Fe standard conditions (25 μM, plain thin arrow), both IRT1 and NRAMP1 contribute to Fe uptake in the epidermis and cortex. Fe ions, which invade the apoplastic space of root cells located outside the casparian strip, become a substrate for the endodermis-located NRAMP1 transporter, thus penetrating the root central cylinder. Either one of the two IRT1 and NRAMP1 transporters is sufficient to carry out Fe acquisition but the simultaneous absence of the two transporters limits plant growth even in condition of Fe-sufficiency. (**iii**) Under high Fe supply (>500 μM, plain wide arrow), a very low affinity acquisition system exists, which can overcome the absence of both IRT1 and NRAMP1 since growth of an *irt1 nramp1* mutants is rescued by 500 μM Fe supply and over. e, epidermis; c, cortex; en, endodermis; cs, casparian strip; p, pericycle; x, xylem.

In conclusion, the main outcome of this study is the finding that NRAMP1 cooperates with IRT1 to take up Fe in roots under replete conditions. A possible scenario for low affinity Fe transport involves parallel symplastic and apoplastic transports in the outer cell layers of the root (Fig. 6). It is well established that under Fe limiting supply, IRT1 takes up Fe into epidermal and cortical cells, hence serving as the main entry point for the symplastic transport of Fe en-route towards the root central cylinder. A parallel pathway using the apoplastic route up to the casparian strip might operate under Fe replete conditions, requiring a transporter at the surface of endodermal cells to penetrate and reach the inner part of the root. Given *NRAMP1* high expression in endodermis^25^,^34^, this transporter is a good candidate to help Fe overcome the apoplastic barrier and penetrate the stele. It seems to be a general feature of NRAMP transporters to transport alternatively Fe and Mn, choosing whichever of the two substrates is the most abundant. This was nicely shown for the Arabidopsis vacuole exporters NRAMP3 and NRAMP4, which transport Fe in the embryo and Mn in mature plants^35^,^36^. We therefore can envision that under Fe replete conditions, the apoplastic pool of Fe surrounding endodermal cells increases, thus outcompeting Mn for transport by NRAMP1. Measuring NRAMP1 transport parameters for Fe should help confirm this hypothesis.

## Methods

### Plant material

The *nramp1-1 irt1-1* double mutant was obtained by crossing *irt1-1* mutant^22^ with *nramp1-1* mutant^25^. Homozygous plants for both *irt1-1* and *nramp1-1* mutations were genotyped by PCR (see Supplementary Table S2 for primer sets and Supplementary Fig. S2). Four independent double mutants were selected and one representative line used in this study.

A population of T-DNA insertion mutants in the Ws background from the Versailles Arabidopsis stock centre collection was screened by PCR with the T-DNA flanking primers (CTGATACCAGACGTTGCCCGCATAA and CTACAAATTGCCTTTTCTTATCGAC) and *NRAMP1* specific primers (CGATGGCGGCTACAGGATCTGGACG and CTCAGTCAACATCGGAGGTAGATACTC), enabling to isolate the mutant EFD257, which carries a T-DNA insertion at the 3′-end of *NRAMP1* first exon, 350 bp downstream the start codon. This line is referred to as the *nramp1-2* allele. *nramp1-2 irt1-1* double mutant was then obtained by crossing *nramp1-2* and *irt1-1* single mutants and genotyped by PCR (see Supplementary Table S2 for primer sets).

*HA*-*NRAMP1* cDNA expressed under the control of a 1 Kb *IRT1* promoter (*pIRT1::NRAMP1* for simplicity) was introgressed into *nramp1-1* and *nramp1-1 irt1-1* mutant backgrounds by crossing *nramp1-1* mutant with *irt1-1* mutant stably transformed with that construct^25^. *nramp1-1* mutants homozygous for the *pIRT1::NRAMP1* transgene, referred to as *nramp1 pIRT1::NRAMP1, and nramp1-1 irt1-1* double mutant homozygous for the *pIRT1::NRAMP1* transgene, referred to as *nramp1 irt1 pIRT1::NRAMP1,* were both selected by PCR (*nramp1-1* mutation), Basta resistance (*irt1-1* mutation) and resistance to hygromycine conferred by the transgene (*pIRT1::NRAMP1*). *irt1 pNRAMP1::IRT1* and *nramp1 pNRAMP1::IRT1* transgenic lines were obtained by floral dipping of *irt1*^37^ and *nramp1-1* plants with *Agrobacterium tumefaciens* strain GV3101 co-transformed with pSOUP and *pNRAMP1::IRT1*-pG0229 (see cloning) and selected based on their resistance to Basta. Three independent lines were selected and one representative line is shown in this study.

### Growth conditions

Mutants and wild type seeds were vertically grown *in vitro* at 21 °C under long days (16 h light/8 h dark). Seeds were surface-sterilized, sown on half-strength Murashige and Skoog medium (½MS, standard conditions) containing 0.8% agar, 1% sucrose, 0.5 g. L^−1^ MES (pH 5.7 with KOH) and stratified two days at 4 °C in the dark. For hydroponic cultures, one week old seedlings grown *in vitro* under the above-mentioned conditions were transferred to a nutrient standard solution (0.264 mM NH_4_NO_3_, 1 mM Ca(NO_3_)_2_, 1.36 mM KNO_3_, 0.386 mM MgSO_4_, 0.152 mM KH_2_PO_4_, 2.6 μM MgCl_2_, 5 μM H_3_BO_3_, 0.08 μM ZnSO_4_, 0.01 μM CuSO_4_, 0.013 μM MoO_3_, 20 μM Mn(SO_4_), 25 μM FeIII(Na)-EDTA) and further grown for the indicated time under short days (8 h light/ 16 h dark, 300 mE.s^−1^, 70% relative humidity, 21 °C). When indicated Fe or Mn were either omitted from the nutrient solution (-Fe and -Mn respectively) or supplied at the concentration mentioned in the legend of the figures. Solutions were changed weekly and the day before harvest.

### Cloning

A 1.65 Kb *NRAMP1* promoter^25^ and a full-length *IRT1* genomic fragment^38^ were PCR-amplified on Columbia genomic DNA with the primer sets listed in Supplementary Table S2 introducing KpnI/EcoRI and EcoRI/PstI restriction sites on the 5′/3′ ends of the respective fragments. The fragments were then ligated into a KpnI/PstI digested pG0229 binary vector to generate pG0229-*pNRAMP1::IRT1*. Positive clones were selected by PCR and sequenced.

### RNA extraction and RT-qPCRs

Total RNA was isolated with Trizol reagent (Invitrogen) according to the manufacturer’s instructions. Reverse transcription was performed with oligo d(T) primers on 1 to 2 μg of total RNA digested with DNAse I (Thermo Scientific) using the RevertAid First Strand cDNA Synthesis Kit (Thermo Scientific) according to the manufacturer’s protocols. The qPCRs were performed using the Roche Light Cycler 480 II instrument with the primer sets listed in Supplementary Table S2 and the SYBR^®^ Premix Ex Taq (Tli RNaseH Plus), Bulk (Takara).

### Histochemical Fe staining

Roots of 15 day-old seedlings grown vertically on ½ MS, were washed 30 min with 2 mM CaSO_4_ and 10 mM EDTA, then rinsed in water before to be incubated for 30 min at room temperature in the Perls solution containing an equal volume of 4% (v/v) HCl and 4% (w/v) potassium-ferrocyanide^39^. For the Perls-DAB staining of sections, roots were first fixed by 30 min vacuum infiltration in a solution containing 2% (w/v) paraformaldehyde, 1% (v/v) glutaraldehyde, 1% (w/v) caffeine in 100 mM phosphate buffer (pH 7) and subsequently incubated for 15 h in the same solution. The samples were washed with 0.1 M Na-phosphate buffer (pH 7.4) three times, and dehydrated 1 h in successive baths of 50, 70, 90, 95, and 100% Ethanol, butanol/ethanol 1:1 (v/v) and 100% butanol. Roots were then embedded in the Technovit 7100 resin (Kulzer) according to the manufacturer’s instructions and thin sections (5 μm) were cut. The sections were deposited on glass slides that were incubated for 45 min in the Perls solution described above. After washing with distilled water, slides were placed in a methanol solution containing 0.01 M NaN_3_ and 0.3% (v/v) H_2_O_2_ for 1 h, and then washed with 0.1 M phosphate buffer (pH 7.4). Slides were then incubated 30 min in a 0.1 M phosphate buffer (pH 7.4) solution containing 0.025% (w/v) DAB (tetrahydrochloride, Sigma, St Louis, Mo, USA), 0.005% (v/v) H_2_O_2_ and 0.005% (w/v) CoCl.6H_2_O (intensification solution). Slides were finally washed in distilled water to stop the reaction and observed under an optical microscope.

### Chlorophyll fluorescence measurements

A kinetic imaging fluorometer (FluorCam FC 800-O, Photon Systems Instruments, Czech Republic) was used to capture chlorophyll fluorescence images and to estimate the maximal quantum yield [*Fv/Fm *= (*Fm−Fo*)*/Fm*] and the effective quantum yield (φ_II_) of the photosystem II. Four week-old plants grown hydroponically under standard conditions (25 μM Fe, 20 μM Mn) were first dark-adapted for 30 min and saturating flashes (0,8 s, 1000 μmol·m^−2^·s^−1^) were then shot to measure the maximum fluorescence. Measurements were performed at room temperature.

### Elemental analysis

Roots and shoots were harvested separately. Roots were first washed 10 min with 2 mM CaSO_4_ and 10 mM EDTA, then washed 3 min with 0.3 mM bathophenanthroline disulphonate and 5.7 mM sodium dithionite and finally rinsed in deionized water. Shoots were rinsed with deionized water. Samples were dried at 80 °C for at least 2 days and mineralized in 7.5% H_2_O_2_, 49% HNO_3_ at 120 °C. Elemental analyses were performed by Micro Plasma Atomic Emission Spectroscopy (Agilent 4200 MP-AES) according to the manufacturer’s recommendations.

## Additional Information

**How to cite this article**: Castaings, L. *et al*. The high-affinity metal Transporters NRAMP1 and IRT1 Team up to Take up Iron under Sufficient Metal Provision. *Sci. Rep.* **6**, 37222; doi: 10.1038/srep37222 (2016).

**Publisher’s note:** Springer Nature remains neutral with regard to jurisdictional claims in published maps and institutional affiliations.

## Supplementary Material

Supplementary Information
