# Supplementary material for: The high-affinity metal Transporters NRAMP1 and IRT1 Team up to Take up Iron under Sufficient Metal Provision
Source: Sci Rep. 2016 Nov 16;6:37222. doi: 10.1038/srep37222 (PMC5110964; doi:10.1038/srep37222)
Supplement: Supplementary Information [file srep37222-s1.pdf]

Online Supplementary Information

The high-affinity metal Transporters NRAMP1 and IRT1 Team up to Take up Iron under Sufficient Metal Provision

Loren Castaings[1], Antoine Caquot[1], Stéphanie Loubet[1] and Catherine Curie[1*]

Supplementary Methods

GUS staining

Seeds from the previously described *pNRAMP1::GUS* [25] line were first surface-sterilized, sown on half-strength Murashige and Skoog medium (½MS, standard conditions) containing 0.8 % agar, 1 % sucrose, 0.5 g.L^{-1} MES (pH 5.7 with KOH) then stratified two days at 4°C in the dark and finally horizontally grown for 15 days under long days (16h days:8h night) at 21°C. Seedlings were harvested, immerged in the staining solution [1 mg.ml^{-1} X-Gluc (5-bromo-4-chloro-3-indolyl-β-D-glucuronide), 2 mM potassium ferrocyanide, 2 mM potassium ferricyanide dissolved in phosphate buffer (50 mM Na_2HPO_4, 50 mM NaH_2PO_4, pH7) 0.1 % Triton X-100] and vacuumed twice 5 min to allow the solution to penetrate the tissues. Coloration was allowed to develop overnight at 37°C. The staining solution was replaced by ethanol 70 % until leaf chlorophyll removal was complete. Seedlings were then rinsed in water and observed under an optical microscope.

Supplementary Tables and Figures

Supplementary Table S1: **Elemental analyses in shoots and roots.**

Fe, Mn, Zn and Cu content in roots and shoots of 4 week-old plants grown in hydroponic conditions in standard medium. Mean ± sd. (n=3 to 4 replicates of 8 to 10 plants each).

	**Fe**		**Mn**		**Zn**		**Cu**	
	**Shoots**	**Roots**	**Shoots**	**Roots**	**Shoots**	**Roots**	**Shoots**	**Roots**
**WT**	89.40 ± 13.6	570 ± 37.12	879.04 ± 70.27	121.97 ± 30.61	44.55 ± 10.66	50.67 ± 19.86	5.05 ± 1.48	8.56 ± 1.74
nramp1-1	100.16 ± 6.05	538.16 ± 26.37	879.39 ± 88.45	154.17 ± 52.75	30.58 ± 2.51	32.24 ± 6.86	3.08 ± 0.3	5.46 ± 0.49
irt1-1	97.85 ± 11.35	658.65 ± 33.80	817.14 ± 25.43	203.95 ± 37.70	34.76 ± 7.38	34.27 ± 7.86	3.58 ± 0.49	6.64 ± 1.04
nramp1-1 irt1-1	**45.40** ± 13.37	**1,883.03** ± 111.07	**545.99** ± 40.66	**58.36** ± 10.24	46.15 ± 6.17	82.44 ± 11.92	4.83 ± 0.92	8.27 ± 1.89

Supplementary Table S2: Primers list.

	Forward	Reverse
**qPCR primers**		
ACTIN	GGTAACATTGTGCTCAGTGGTGG	AACGACCTTAATCTTCATGCTGC
NRAMP1	CGGAACTTATGCTGGACAAT	AGAAGAGGAACCAACGCAAA
IRT1	CGGTTGGACTTCTAAATGC	CGATAATCGACATTCCACCG
FRO2	GCGACTTGTAGTGCGGCTATG	CGTTGCACGAGCGATTCTTG
FER1	TCGTTGAGAGTGAATTTCTGG	ACCCCAACATTGGTCATCTG
**Genotyping**		
nramp1-1	GCGTGGACCGCTTGCTGCAACT(LB1b)	GTCAACATCGGAGGTAGATACTCTACATGGTAACTGC
	GCGGGGCTGTTTGTAATGCC	GTCAACATCGGAGGTAGATACTCTACATGGTAACTGC
nramp1-2	CTACAAATTGCCTTTTCTTATCGAC (Tag5)	CGATGGCGGCTACAGGATCTGGACG
	GCTGCTACAGGATCTGGACGTTCTCAATTC	GTCAACATCGGAGGTAGATACTCTACATGGTAACTGC
irt1-1	GTTAAGCCCATTTGGCGATAATCGACATTC	CTACAAATTGCCTTTTCTTATCGAC
	CACACAAACATTAAACAATC	GAAAAAGCAGCAAAAGTTTTATTTA
**Cloning**		
pNRAMP1	GGGCACGGTACCAATCATGTCTTAAGCTTC	GGGCACGAATTCCGCCGTCTCTCTCTTCCTA
IRT1 full-lenght	GGGCACGAATTCCACACAAACATTAAACAATC	GGGCACCTGCAGGAAAAAGCAGCAAAAGTTTTATTTA

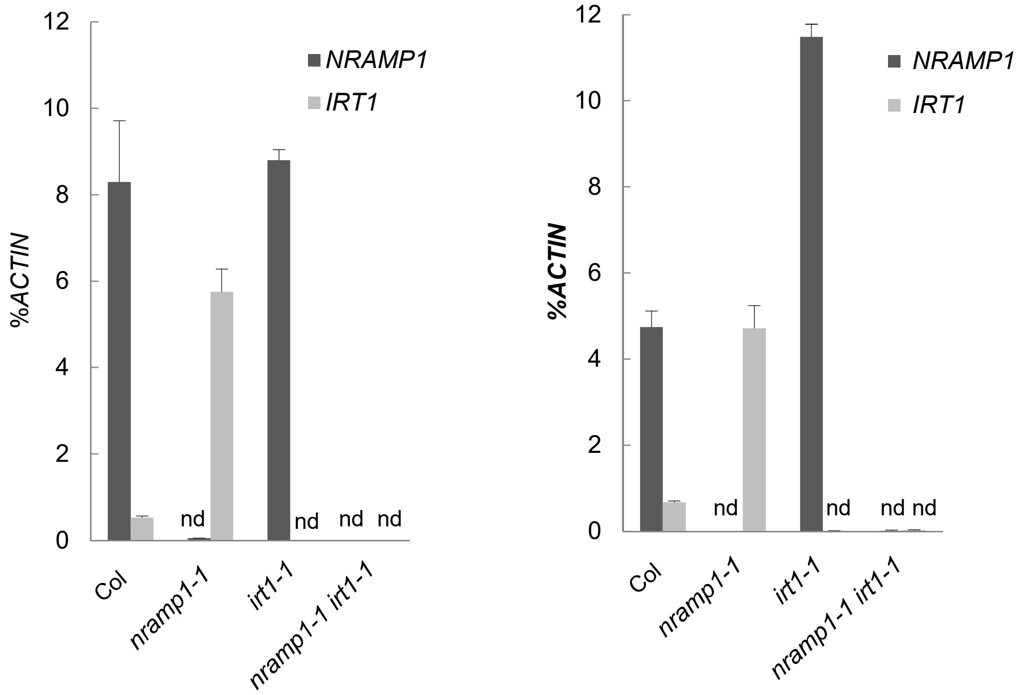

Supplementary Figure S1: Expression analysis of *IRT1* and *NRAMP1* genes in roots. RT-qPCR was performed on root RNA from wild-type, *nramp1-1*, *irt1-1* and *nramp1-1 irt1-1 plants* grown in hydroponics for 4 weeks under standard conditions (25 µM Fe, 20 µM Mn). Two independent experiments are shown (left and right). Transcripts levels are expressed relative to those of the reference gene *ACTIN* ± sd. (n= 3 technical replicates). nd, non-detectable.

a

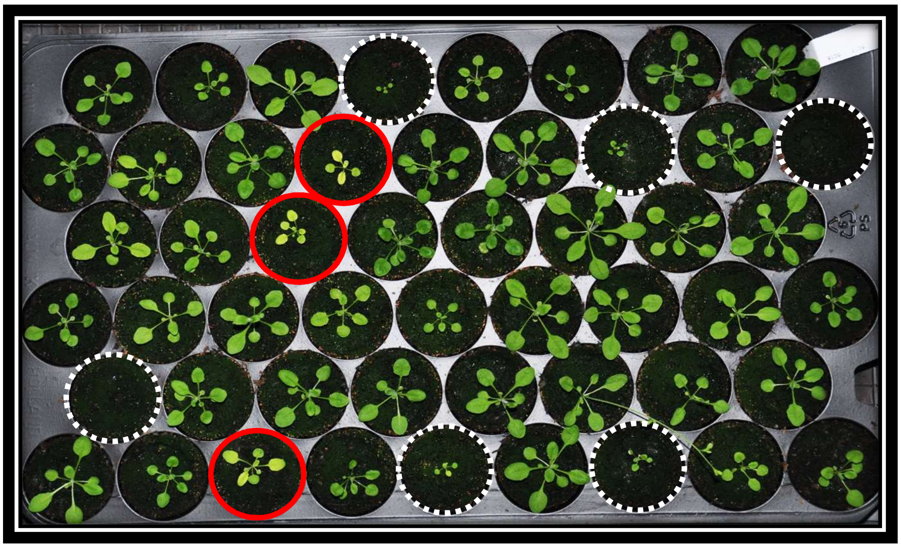

b

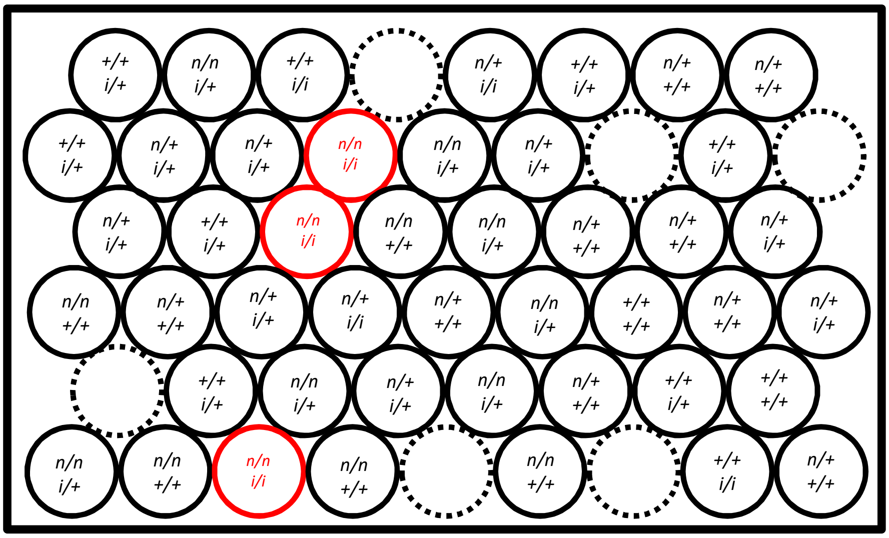

Supplementary Figure S2: Phenotyping and genotyping of the F2 progeny of a cross between *nramp1-1* and *irt1-1*.

Plants were supplied with Fe through irrigation with 500 mg/L Fe-EDDHA as well as spraying of the leaves every other day with 200 µM Fe-citrate. **(a)** picture of the plants 20 days after sowing. Three out of 45 F2 progeny plants, *i.e.* the expected 1 to 16 segregation ratio for two independent loci, showed slow growth and a strong chlorosis (circled in red) and were the only ones of this population to be genotyped as being homozygous for both *nramp1-1* and *irt1-1* mutations. **(b)** genotyping results of the plants shown in (a). Dashed circles indicated dead seedlings or seedling excluded from genotyping because of a delay in germination. Wild type allele (*+*), *irt1-1* mutant allele (*i*) and *nramp1-1* mutant allele (*n*).

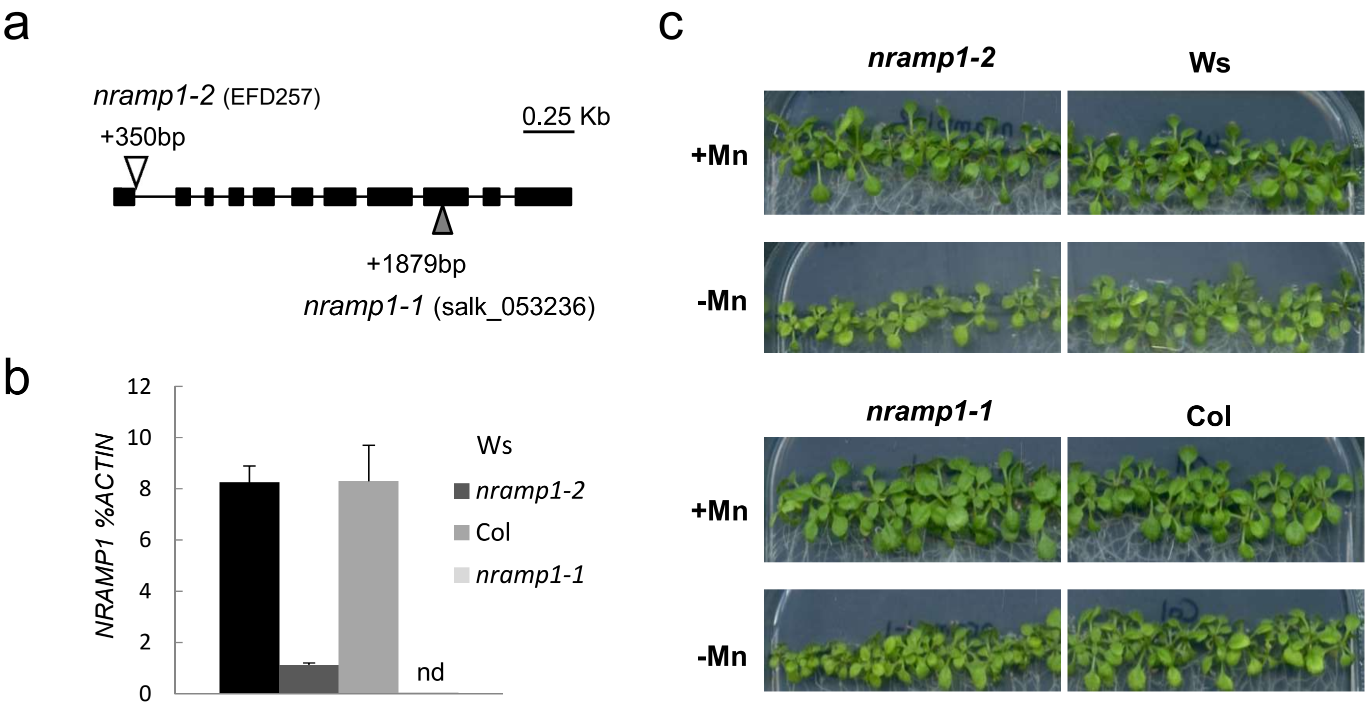

Supplementary Figure S3: The *nramp1-2* knock-down allele is hypersensitive to Mn deficiency. (**a**) Position of T-DNA insertions in the *NRAMP1* gene for the *nramp1-1* (grey triangle) and *nramp1-2* (white triangle) alleles. (**b**) *NRAMP1* expression in roots of *nramp1-1* and *nramp1-2* mutants compared to their respective wild type parent. Plants were grown 4 weeks in hydroponic standard conditions (25 µM Fe, 20 µM Mn). Transcripts levels were measured by qRT-PCR relative to those of the reference gene *ACTIN* ± sd. (n= 3 technical replicates). nd, non-detectable. (**c**) Shoot growth phenotype of 14 day-old *nramp1* mutants grown *in vitro* with (+Mn) or without Mn (-Mn) in the medium as compared to their respective wild-type parent.

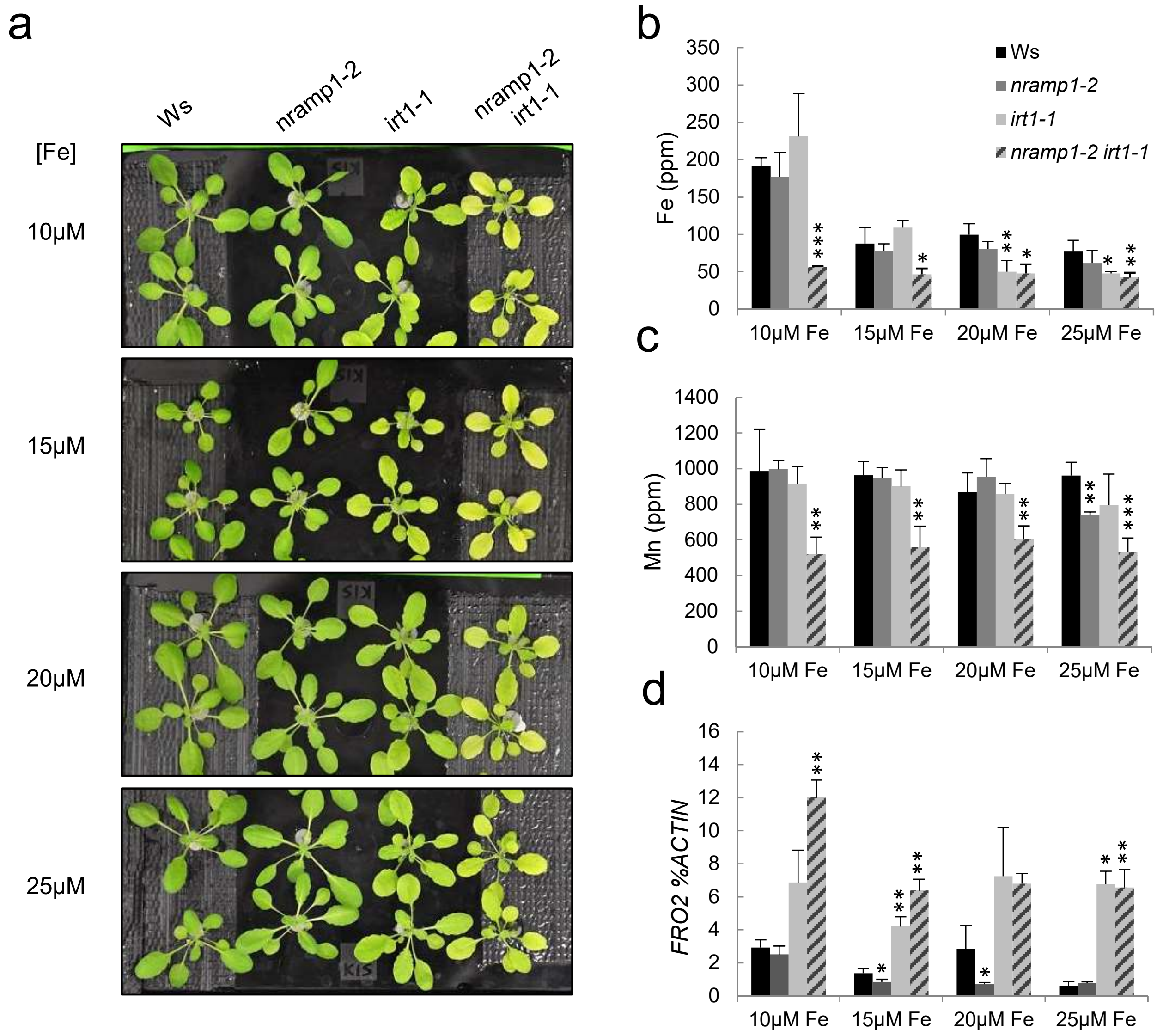

Supplementary Figure S4: The second allele *nramp1-2 irt1-1* is also hypersensitive to Fe limitation. (**a**) 28 day-old plants grown in hydroponic medium containing a range of Fe-EDTA concentrations. (**b**) and (**c**) MP-AES measurement of Fe (**b**) and Mn (**c**) content in shoots of the plants shown in (**a**). Means ± sd. (n= 2 to 3 replicates of 3 plants each). (**d**) *FRO2* expression in roots of the plants shown in (**a**). Transcripts levels were measured by qRT-PCR relative to those of the reference gene *ACTIN* ± sd. (n= 2 to 3 replicates of 3 plants each). Panels (**b**), (**c**) and (**d**) share the colour-code legend. Asterisks indicate values statistically different from the Ws wild-type plants grown at the same Fe concentration (Student T-test, * P-value <0.1, **P-value <0.05, ***P-value < 0.005).

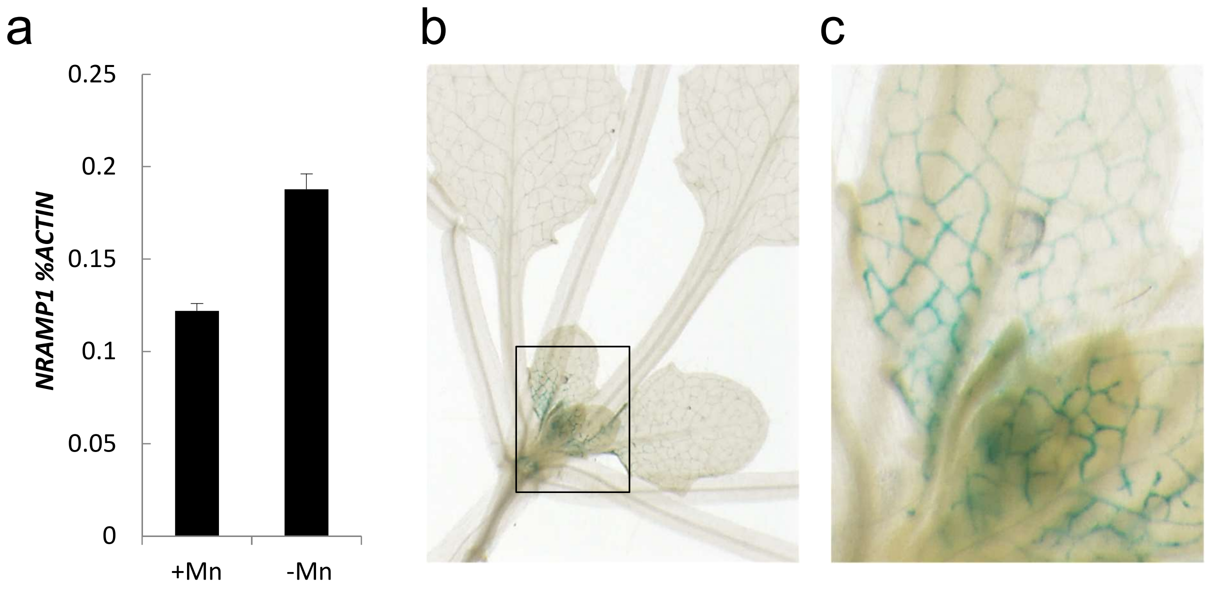

Supplementary Figure S5: *NRAMP1* expression in shoots. (**a**) *NRAMP1* expression in shoots of 15 day-old seedlings vertically grown *in vitro* in standard conditions (+Mn) or in the absence (-Mn) of Mn in the medium. Transcripts levels were measured by qRT-PCR relative to those of the reference gene *ACTIN* ± sd. (n=3 replicates of 8 plants each). (**b**) GUS staining of a 15 day-old *pNRAMP1::GUS* transgenic line grown *in vitro* in standard conditions. (**c**) Magnification of the young leaves shown in (**b**).

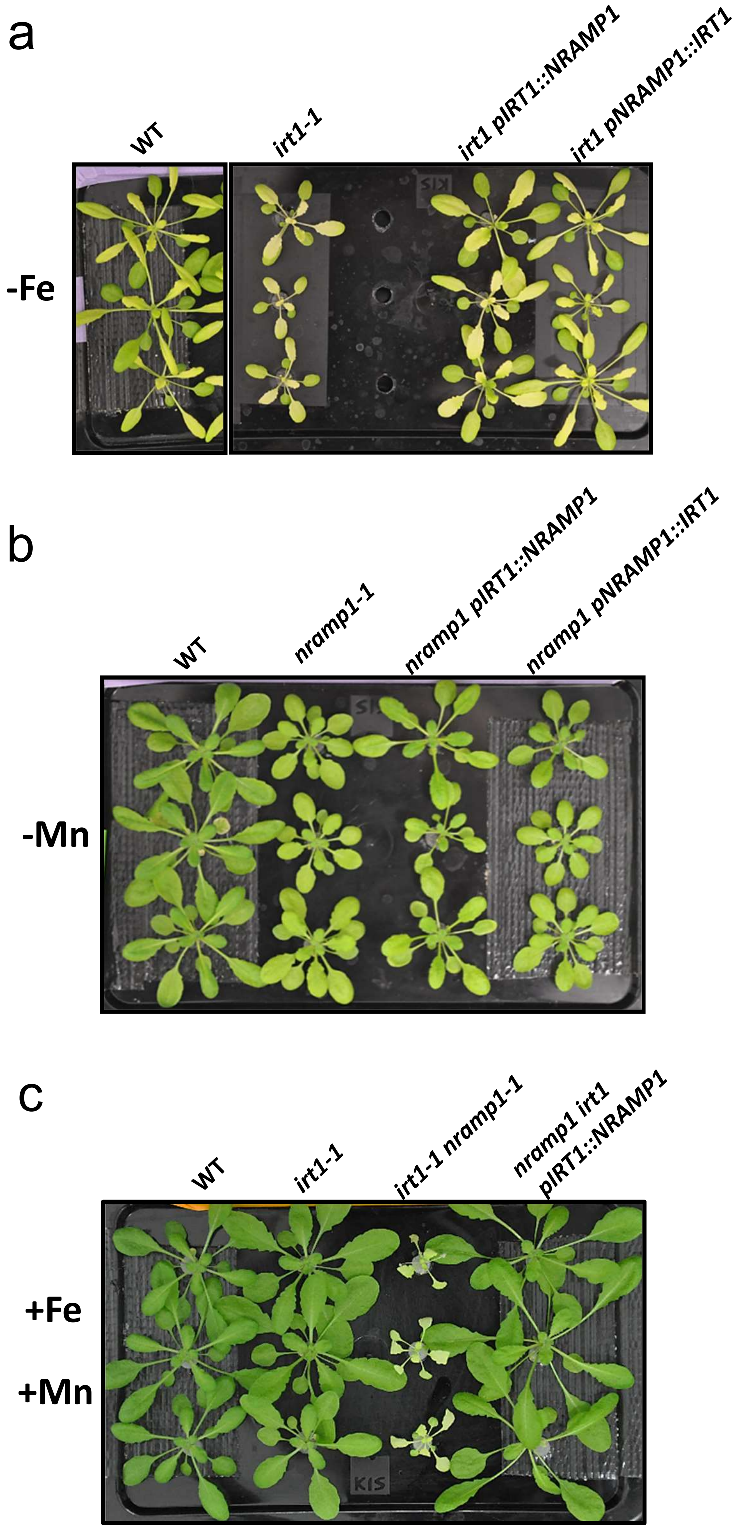

Supplementary Figure S6: Phenotypic complementation of *nramp1-1, irt1-1* and *nramp1-1 irt1-1* mutants by promoter swap constructs. (a) Complementation of *irt1-1* hypersensitivity to iron deficiency by promoter swap constructs. Plants were grown 2 weeks in standard hydroponic conditions and one additional week without added iron (-Fe). **(b)** Complementation of *nramp1-1* hypersensitivity to manganese deficiency by promoter swap constructs. Plants were grown for 3 weeks in hydroponic conditions without added manganese (-Mn). **(c)** Complementation of *nramp1-1 irt1-1* growth defect and chlorosis by *pIRT1::NRAMP1*. Plants were grown for 3 weeks in hydroponic standard conditions (+Fe +Mn).

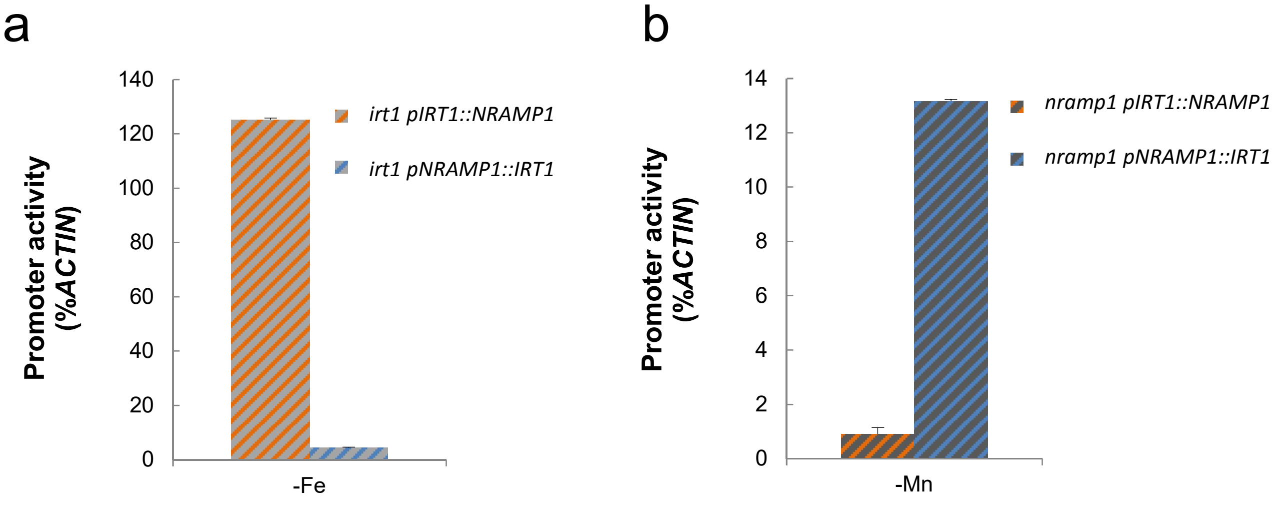

Supplementary Figure S7: Activity of the IRT1 and NRAMP1 promoters in the promoter swap lines. Expression level of *IRT1* and *NRAMP1* produced by the promoter-swap constructs *pNRAMP1::IRT1* and *pIRT1::NRAMP1,* respectively, in roots of the *irt1* **(a)** or the *nramp1* **(b)** mutant. Plants were (a) grown 2 weeks hydroponically under standard condition, then Fe-starved for one additional week (-Fe) or (b) grown for 3 weeks under Mn starvation (-Mn). Transcripts levels were measured by qRT-PCR relative to those of the reference gene *ACTIN* ± sd. (n=3 biological replicates of 3 to 6 individuals).
